# The Utility of Nuclear Imaging in Hypertrophic Cardiomyopathy: A Narrative Review

**DOI:** 10.3390/jcm14072183

**Published:** 2025-03-22

**Authors:** Sukruth Pradeep Kundur, Ali Malik, Sanjay Sivalokanathan

**Affiliations:** 1 Faculty of Life Sciences and Medicine, King’s College London, London WC2R 2LS, UK; ali.t.malik@kcl.ac.uk; 2 Mount Sinai Health System, Icahn School of Medicine at Mount Sinai, New York, NY 10029, USA

**Keywords:** hypertrophic cardiomyopathy, nuclear imaging, sudden cardiac death, PET, SPECT, inherited cardiac diseases, risk stratification

## Abstract

Hypertrophic cardiomyopathy (HCM) is the most common genetically inherited cardiac condition, characterized by clinical heterogeneity and a significantly increased risk of adverse cardiovascular outcomes, including sudden cardiac death. Current diagnostic methods primarily use echocardiography, often supplemented by cardiovascular magnetic resonance imaging (MRI), to assess the clinical profile of the disorder. Effective risk stratification protocols are essential for managing patients with HCM. These models rely on patient histories, imaging findings, and genetic information to evaluate the prognosis. Important factors in risk assessment include severe left ventricular hypertrophy (>30 mm), late gadolinium enhancement (>15%), or the presence of an apical aneurysm. However, these imaging techniques may lack sensitivity when it comes to detecting ischemia or microvascular dysfunction. Nuclear imaging methods, such as single-photon emission computed tomography (SPECT) and positron emission tomography (PET), offer a more comprehensive analysis of HCM. They provide valuable insights into the complex physiological mechanisms underlying the disease and facilitate early detection of functional abnormalities. This is particularly important for identifying high-risk phenotypes and understanding associated risk factors that may increase morbidity or mortality. This narrative review focuses on the role of nuclear imaging in the context of hypertrophic cardiomyopathy.

## 1. Introduction

Hypertrophic cardiomyopathy (HCM) is recognized as the most prevalent genetic cardiovascular condition. This disorder is predominantly inherited in an autosomal dominant fashion, commonly arising from mutations in the β-myosin heavy chain (MYH7) or myosin-binding protein C (MYBPC3) genes, which encode cardiac sarcomeric proteins [1]. HCM is characterized by asymmetric hypertrophy of the left ventricle, with a wall thickness of 15 mm or greater, and it occurs in the absence of abnormal loading conditions [2]. Most recently, it has become apparent that factors such as body size, age, and gender may impact the accurate determination of maximal wall thickness in the diagnosis of HCM [3]. Importantly, it remains a significant identifiable cause of sudden cardiac death (SCD) in individuals under the age of 35, with a notably high mortality rate among athletes [4]. Invariably, it is associated with a range of clinical consequences, ranging from the development of heart failure to SCD. Given the grave implications of this disorder, particularly since it is unpredictable, it is imperative to establish robust diagnostic frameworks to effectively assess and diagnose the sequelae of HCM.

Once considered a disease with high morbidity, advances in diagnostics and therapy have enabled early detection as well as improved survival. However, there remains a pressing need for more sophisticated imaging modalities that extend beyond structural assessments. Such innovations would enable an improved evaluation of disease progression, risk stratification, and therapeutic response. This evolution would further support the transition from traditional medical and interventional approaches to more personalized treatment regimens, including targeted pharmacotherapy, gene therapies, and surgical procedures.

Nuclear imaging is a non-invasive tool that provides functional insights into cardiovascular disease. It facilitates risk stratification and classification of pathophysiological processes. Radioactive tracer molecules are administered to target specific anatomical structures, which subsequently emit radiation upon binding, enabling external detection and image acquisition. The two predominant modalities employed in this field are positron emission tomography (PET) and single-photon emission computed tomography (SPECT). However, the role of PET and SPECT in HCM has traditionally been viewed as limited.

This review aims to provide a comprehensive examination of the role of nuclear imaging in HCM, including the evaluation of current advancements in various nuclear imaging modalities and their applications in clinical practice. Furthermore, this review discusses potential limitations within the field of nuclear medicine and outlines future directions, highlighting the significant role that nuclear imaging may play as diagnostic and management strategies for HCM continue to evolve.

## 2. Background

### 2.1. Genetic Basis of Disease

Approximately 30 to 40% of cases of HCM exhibit a pathogenic genetic variant. A significant proportion of affected individuals inherit the condition in an autosomal dominant fashion [5]. Aside from the most common MYH7 and myosin-binding protein C (MYBPC3) genes associated with HCM, other variants implicated include TNNT2, TNNI3, and TPM1, as well as weaker associations with mutations in ACTC1 (cardiac α-actin), MYL2 (myosin light chain 2), MYL3 (myosin light chain 3), and CSRP3 (Cysteine and Glycine Rich Protein 3) [6,7].

### 2.2. The Pathophysiology of HCM

Sarcomeric mutations impair myofilament structure and increase calcium sensitivity, leading to hypercontractility [8]. Furthermore, abnormal ATP utilization results in dysfunctional metabolism and ineffective contractions, thereby inducing cellular stress [9]. These factors may activate intrinsic hypertrophic signaling pathways, leading to pathological growth of myocardial tissue. The consequences of hypertrophy include dynamic left ventricular outflow tract (LVOT) obstruction, mitral regurgitation, diastolic dysfunction, and microvascular ischemia. The degree of LVOT obstruction permits the classification of HCM into three subtypes: nonobstructive (less than 30 mmHg at rest and during stress), labile (less than 30 mmHg at rest and 30 mmHg or greater with stress), and obstructive (30 mmHg or greater at rest and during stress) [10].

In patients with HCM, myocardial ischemia is also prevalent, often associated with microvascular abnormalities and compromised coronary flow reserve. An imbalance between oxygen supply and demand activates intracellular pathways that lead to myocardial fibrosis [11]. Subsequent widespread fibrosis and abnormal calcium-myofilament coupling may result in diastolic dysfunction. The increased stiffness of the ventricle hinders relaxation and diastolic function, consequently raising intracavity-filling pressures [12]. Parameters such as fibrosis and ischemia serve as critical prognostic markers for HCM, yet they are frequently underappreciated in clinical guidelines. To gain further insights, nuclear imaging can be utilized to assess myocardial perfusion and fibrosis [13].

### 2.3. Principles of Diagnosis and Stratification of Risk

The diagnosis of HCM is challenging since there is a wide spectrum in its clinical presentation. Beyond comprehensive clinical history taking, with a focus on family history, the initial diagnostic algorithm is followed by an electrocardiogram (ECG). Transthoracic echocardiography serves as the primary imaging modality for the diagnosis and evaluation of HCM. The principal diagnostic criterion is the thickening of the myocardium, defined as a measurement of 15 mm or greater. Secondary findings in HCM may include systolic anterior motion of the mitral valve and LVOT obstruction. Stress echocardiography serves a vital role in evaluating dynamic changes in left ventricular outflow obstruction that occur following physiological provocation.

Cardiovascular magnetic resonance (CMR) imaging is considered the gold standard for HCM assessment, as it allows for precise measurement of left ventricular wall thickness, examination of mitral valve abnormalities, and characterization of myocardial tissue abnormalities, including fibrosis through late gadolinium enhancement (LGE) [14]. Nevertheless, a multimodal approach remains crucial for comprehensively evaluating HCM morphology. Such an approach aids in assessing prognosis, understanding pathophysiological classifications, and informing treatment strategies. Upon diagnosis, patients can undergo risk stratification and prognostic evaluation, which are influenced by imaging parameters as well as key factors from the patient’s medical history.

### 2.4. Management of HCM

The management strategies employed in HCM have significantly evolved over recent decades, encompassing lifestyle modifications as well as medical and surgical interventions. The primary objectives of medical management are to control symptoms and optimize the physiological sequelae of HCM. In patients diagnosed with obstructive HCM, treatment may involve the use of beta blockers and calcium channel blockers, which are frequently combined with disopyramide to achieve a negative inotropic effect. This combination aims to reduce myocardial oxygen demand, enhance diastolic filling, and alleviate outflow tract obstruction. In addition, antiarrhythmics may be prescribed to patients experiencing atrial fibrillation or ventricular arrhythmias. The management of other complications, including heart failure, adheres to established clinical guidelines, and in severe cases, heart transplantation may be considered [15].

Surgical management aims to alleviate the disease burden and is typically reserved for patients exhibiting severe outflow tract obstruction, defined as greater than 50 mmHg. The gold standard for surgical intervention remains septal myectomy, aimed at relieving the obstructive burden, with a subsequent decrease in myocardial mass [16]. An effective alternative is alcohol septal ablation [17]. Nuclear imaging has been utilized in research to evaluate patients’ eligibility for interventional management and to assess the success of surgical procedures by monitoring improvements in both structural and physiological parameters following surgical intervention. Furthermore, patients identified as having a high risk of sudden cardiac death (SCD), based on personal history or prognostic scoring systems, may be offered the implantation of an implantable cardioverter defibrillator (ICD) [18]. Emerging therapies, such as myosin inhibitors (e.g., mavacamten or aficamten), ninerafaxstat, as well as gene therapies, show considerable promise; however, they remain in developmental stages and require extensive testing [17].

## 3. Methods

A systematic search following Preferred Reporting Items for Systematic Reviews and Meta-Analyses guidelines was conducted using PubMed, Embase, and Scopus databases for studies focusing on the applications of nuclear imaging in hypertrophic cardiomyopathy (Supplementary Figure S1). Key search terms included “hypertrophic cardiomyopathy”, “nuclear imaging”, “positron emission tomography”, “PET”, “single-photon emission computerized tomography”, and “SPECT”. Additional articles were identified through cross-referencing.

The studies included in this review were selected based on their relevance to nuclear imaging in HCM, with an emphasis on diagnosis, risk stratification, and management of the condition. Original research articles, encompassing reviews, observational studies, and both prospective and retrospective trials, as well as pertinent conference abstracts, were incorporated. In contrast, descriptive case reports and animal studies were excluded from consideration. The primary criteria for the preliminary exclusion of studies during the initial search phase comprised a focus on other cardiac conditions, the application of non-nuclear imaging modalities, and publications in languages other than English.

Following an initial screening of titles and abstracts for relevant studies, full-text articles were assessed based on their objectives, clinical significance, and novelty. Given the extensive body of literature available in this domain, studies published from 2000 onward were included to delineate the key principles of nuclear imaging in HCM. More recent studies were summarized for their clinical applicability. Due to the narrative format of this review, a formal risk of bias assessment was not conducted; however, a critical analysis of the studies was undertaken, and an effort was made to provide a balanced discussion that encompasses both positive and negative findings concerning the role of nuclear imaging in HCM. Moreover, quantitative synthesis and meta-analysis were deemed inappropriate due to the variability among studies and the research’s broader scope. Accordingly, the primary objective remained focused on thematic analysis.

## 4. Results

A comprehensive search of the literature identified 219 articles relevant to the focus of this review, and after careful consideration and comprehensive eligibility assessment, 63 studies were incorporated into this review for data extraction. SPECT and PET imaging modalities featured heavily in the literature identified, allowing the analysis to be focused on these two types of nuclear imaging. Studies were further subclassified into their utility in identifying and characterizing various underlying pathophysiological processes in HCM and their clinical relevance. Data were extracted from relevant study parameters, and the results were summarized in Table 1 (SPECT) and Table 2 (PET).

### 4.1. Nuclear Imaging Modalities in HCM

Nuclear imaging modalities can play a vital role in developing an understanding of the contributory pathological processes and, thus, managing HCM. As aforementioned, diverse underlying processes such as left ventricular hypertrophy, microvascular dysfunction, and myocardial fibrosis characterize HCM, presenting challenges in prognosis and treatment. Traditional imaging techniques such as magnetic resonance imaging (MRI) and echocardiography offer a precise anatomical assessment of the condition and identification of complications, yet often fall short in characterizing the dynamic pathophysiological processes influencing the risk of adverse outcomes.

Nuclear imaging modalities such as SPECT and PET provide insights into the metabolic, functional, and perfusion dynamics of the myocardial tissue, allowing the detection of subtle ischemic changes, metabolic abnormalities, and quantification of fibrosis—which are all factors directly associated with higher risk of complications such as heart failure and SCD. Additionally, processes such as autonomic nervous dysfunction, myocardial energetics, and metabolic abnormalities can be identified by studying patterns of specific PET and SPECT radiotracer uptake.

SPECT historically employed Thallium-201 (201-Tl) as the primary radiotracer to assess myocardial perfusion, but radiotracers such as technetium-99m (99m-Tc) sestamibi and tetrofosmin are preferred due to their favorable technical properties. In contrast, PET has superior sensitivity and image resolution, employing radiotracers such as 13N-ammonia (13N-NH3) and 15-O labeled water in perfusion imaging and 18F-fluorodeoxyglucose (18F-FDG) in quantifying metabolic dysfunction. Both 11C-acetate and 13C-acetate have also been used in PET-CT studies to evaluate myocardial oxygen consumption and energetics. Tracers such as 11C-hydroxyephedrine and 18F-fluorodopamine detect impaired catecholamine activity, which is similar to the use of 123I-metaiodobenzylguanidine (MIBG) SPECT in imaging sympathetic innervation. Novel radiotracers targeted to proteins, such as fibroblast activation protein inhibitors (FAPI), are emerging to detect dynamic molecular changes in the myocardium preceding fibrosis. These advanced imaging techniques are valuable tools to aid risk stratification and monitoring of patients with HCM. The following section explores the clinical applications of nuclear imaging modalities in the assessment of HCM, supported by key findings in the literature. Table 1 and Table 2 offer an overview of key studies investigating the utility of SPECT and PET in assessing HCM, respectively.

### 4.2. Characterizing Myocardial Ischemia

A common symptom of HCM patients is chest pain, which may be elucidated by the underlying mechanisms involving the imbalance between coronary dysfunction and the elevated metabolic demands of the hypertrophied myocardium [82]. Myocardial ischemia is interrelated with adverse consequences in HCM, which include necrosis and cell death, fibrosis, and left ventricular dilatation. The severity of dysfunction can cause debilitating clinical consequences that impair their quality of life. However, there are discrepancies in understanding myocardial ischemia in HCM. The etiology of myocardial perfusion abnormalities has been speculated to be linked to microstructural abnormalities, as well as macrostructural features such as LVOT obstruction.

#### 4.2.1. Myocardial Perfusion Imaging with SPECT

Whilst myocardial perfusion imaging with SPECT is well established within the literature and clinical practice across a range of cardiovascular diseases, it has been used to identify numerous underlying abnormalities in the pathophysiology of HCM with clinical implications for adverse outcomes and early detection. SPECT myocardial perfusion imaging (MPI) with 99m-Tc or 201-Tl tracers allows the identification of both fixed and reversible perfusion defects within the myocardium [19,83]. A fixed perfusion defect indicates a region of scar due to fibrosis, often correlating with LGE on cardiac MRI. In contrast, reversible perfusion defects are typically due to areas of transient ischemia from microvascular dysfunction. An early study by Sugihara et al. (1998) examined exercise-induced perfusion abnormalities among patients undergoing Tc-99m tetrofosmin SPECT at 30- and 180-minute intervals post-exercise. Exercise-induced regional perfusion defects were present in 68.2% (30 out of 44) of the subjects, with reverse redistribution observed in 36 patients [84]. Reverse redistribution is characterized by an initially normal-appearing myocardium during stress, with an abnormal radiotracer uptake at rest, predominantly located in the anterior septal wall. This pattern may indicate persistent myocardial injury, microvascular dysfunction, or subendocardial ischemia rather than traditional stress-induced ischemia. The differentiation between fixed and reversible defects is essential for appropriate clinical management. Patients with fixed defects may require coronary evaluation, as it may suggest concomitant ‘macrovascular’ ischemic disease. In contrast, those with reversible defects could benefit from early risk assessment and medical management to prevent the progression of permanent myocardial injury and ventricular dysfunction. The characterization of both fixed and reversible perfusion abnormalities using SPECT imaging can hold significant prognostic value as the extent and severity of these defects correlate with adverse cardiovascular outcomes, including heart failure, arrhythmias, and increased mortality risk [85]. Complications of HCM have been linked with the presence of fixed perfusion defects [23], with patients having a higher number of perfusion abnormalities being found to have higher rates of cardiac deaths [27]. Additionally, a SPECT MPI study with a cohort of 26 patients established methods for indexing subendocardial ischemia and potentially predicting left ventricular cavity dilation [25].

#### 4.2.2. PET and Myocardial Blood Flow

PET has demonstrated significant utility in identifying patterns of underlying microvascular dysfunction. In patients with HCM, regions exhibiting the most pronounced hypertrophy often show compromised myocardial blood flow. Earlier studies conducted in the field support the hypothesis for microvascular dysfunction influencing the progression to myocardial fibrosis and the utility of PET scans with radiotracers such as oxygen-15 water and 13N-NH3 ammonia for detecting subtle patterns of myocardial ischemia correlated with contractile dysfunction [52] and fibrosis [55]. Patients with reduced blood flow have been consistently found to be at higher risk of suffering from adverse consequences of HCM [43,46,47,59,66].

Subendocardial ischemia is a key characteristic of microvascular dysfunction, which plays a crucial role in the progression of left ventricular cavity dilatation (LVCD). Various parameters, including resting and stress myocardial blood flow (MBF), MBF reserve, LVCD, and transmural perfusion gradients (TPG), have been extensively studied utilizing 13N-NH3 PET, typically conducted with hybrid PET-CT imaging. Importantly, employing 13N-NH3 radiotracers facilitates the quantification of both global and regional myocardial blood flow abnormalities, thereby enabling the detection and localization of microvascular dysfunction. Research has indicated that impaired MBF and myocardial flow reserve following stress exercise serve as independent predictors of unfavorable outcomes in HCM patients. In a study by Bravo et al., 13N-NH3 PET/CT with dipyridamole stress was employed to assess myocardial perfusion differences through absolute flow quantification in patients with and without LVOT obstruction [53]. The findings revealed no significant correlation between obstructive HCM and augmented myocardial ischemia; instead, the study concluded that maximal wall thickness emerged as the most reliable predictor of myocardial blood flow [53]. Subtle changes such as endocardium/epicardium blood flow with 15O-water PET were lower in patients who developed non-sustained ventricular tachycardias in a 25-person cohort study [70]. Specific mutations in sarcomeric myofilament genes have also been linked with lower stress myocardial blood flow levels than genotype-negative patients, identifying a potential link for further investigation between genetics and structural changes [49]. Additionally, patients who developed pulmonary hypertension, a significantly adverse consequence of HCM, had reduced myocardial flow reserve and globally reduced stress MBF [67,68].

Moreover, PET has demonstrated the ability to identify decrements in subendocardial perfusion during dipyridamole stress, underscoring its significant contribution to transient LVCD. In a cohort of 104 HCM patients, the assessment of the LVCD index (the ratio of LV volume during stress to LV volume at rest, with a threshold of > 1.13) and the evaluation of transmural perfusion gradients (subendocardial MBF to subepicardial MBF) revealed comparable transmural perfusion gradients in patients with and without LVCD at rest [60]. However, patients exhibiting LVCD demonstrated diminished hyperemic subendocardial MBF during pharmacological stress. This finding suggests that the subendocardial flow does not increase sufficiently under stress conditions, resulting in an imbalance in transmural perfusion gradients and supporting the hypothesis of widespread perfusion abnormalities in HCM. In addition, the study demonstrated that LGE was more prevalent among patients with LVCD, indicating its potential role as an adverse risk factor [60]. Furthermore, a subsequent investigation conducted by Sciagra et al. utilizing 13N-NH3 PET imaging in conjunction with dipyridamole stress demonstrated that patients with impaired left ventricular ejection fraction (LVEF) experienced more pronounced subendocardial ischemia and reduced transmural perfusion gradients during stress [64]. Specifically, the transmural perfusion gradients measured were 1.20 ± 0.39 in patients with impaired LVEF compared to 1.40 ± 0.43 in those with preserved LVEF (*p* < 0.0001) [64].

In supplementary PET studies, LVCD has been explored as an indirect marker of microvascular dysfunction, particularly concerning subendocardial ischemia. Lu et al. (2020) reported that 51% (*n* = 55/108) of their cohort of patients with hypertrophic cardiomyopathy (HCM) demonstrated LVCD on PET during vasodilator stress; however, only one of these patients exhibited evidence of LVCD during exercise-induced stress echocardiography [69]. The cohort that tested positive for PET-LVCD exhibited a lower LVEF during vasodilator stress (43% ± 13% versus 52% ± 11%), greater left ventricular hypertrophy, and significantly diminished myocardial flow reserve (2.20 ± 0.60 versus 2.52 ± 0.80, *p* = 0.02) compared to those without LVCD [69]. Similarly, Bravo et al. (2016) found that patients with HCM who exhibited LVCD on PET imaging had greater left ventricular thickness but lower peak myocardial blood flow (MBF) (1.66 ± 0.42 versus 2.07 ± 0.46 mL/minute/g; *p* < 0.0001) and reduced coronary flow reserve (1.90 ± 0.46 versus 2.46 ± 0.69; *p* < 0.0001) [58]. Additionally, regional myocardial perfusion abnormalities were identified in 31 out of 32 LVCD-positive patients, while only 12 out of 29 LVCD-negative patients displayed similar abnormalities (*p* < 0.0001) [58]. This emphasizes the clinical significance of PET in evaluating perfusion abnormalities and suggests its potential utility as a method for risk stratification.

### 4.3. Metabolic and Functional Imaging

Metabolic dysfunction is a significant characteristic of HCM that plays a crucial role in the progression of the disease. In hypertrophied myocardial segments, abnormal energetic properties are evident, leading to inefficient adenosine triphosphate (ATP) utilization, contributing to energetic insufficiency, ischemia, and fibrosis [61]. HCM is associated with an increased dependence on anaerobic glycolysis within cardiac muscle, which predisposes the myocardium to dysfunction and increases susceptibility to ischemia, even in the absence of obstructive coronary artery disease [9]. PET imaging utilizing 18F-FDG has traditionally been employed to identify cells exhibiting abnormally high metabolic activity, as evidenced by elevated glucose uptake, and is commonly used to detect metastatic tumors [86]. This principle can similarly be applied to HCM; heterogeneous regions exhibiting abnormal FDG uptake may indicate ischemic areas susceptible to complications such as inflammation, necrosis, and fibrosis. Although further definitive studies are necessary utilizing this radiotracer, 11C-acetate PET has garnered attention for its usefulness in evaluating oxidative myocardial metabolism by tracing oxidative metabolism within the tricarboxylic acid (TCA) cycle. The clearance rate of 11C-acetate from the myocardium serves as a marker for myocardial oxygen consumption and oxidative efficiency, both of which are critical in understanding the pathophysiology of HCM [70].

Clinical investigations, such as the study conducted by Aoyama et al., demonstrate that 18F-FDG uptake is predominantly localized to hypertrophied myocardium, likely reflecting increased metabolic demands [61]. In patients with the non-obstructive variant, 18F-FDG uptake is confined to the hypertrophied myocardial segments, whereas in obstructive HCM, the accumulation of 18F-FDG extends beyond the regions of hypertrophy. When correlating patterns of LGE observed on cardiac MRI with metabolic activity in non-obstructive HCM, areas exhibiting FDG uptake may demonstrate LGE, thereby reinforcing the association between metabolic abnormalities and replacement fibrosis. Conversely, in obstructive HCM, non-hypertrophied segments displaying 18F-FDG uptake may not correspond with LGE findings, suggesting that metabolic remodeling might precede structural fibrosis [61]. Furthermore, it has been suggested that reduced 18F-FDG uptake, particularly in the anterior and apical interventricular septum, may indicate tissue necrosis [87]. Collectively, these findings emphasize the prognostic value of 18F-FDG PET in stratifying disease severity and identifying regions at risk for fibrotic transformation. In addition, radionuclide ventriculography (or angiography) may be utilized for volumetric and functional assessment. A peak filling rate < 2.5 end-diastolic volume/s and a time-to-peak filling rate > 180 ms are considered abnormal parameters in HCM [88].

Dual isotope SPECT with 201-thallium and 123I-Beta-methyl iodine phenylpentadecanoic acid (BMIPP) has also been used to study mismatches between myocardial metabolism and perfusion. As 123I-BMIPP is a fatty acid analog, it can be a valuable marker of energy balance. Whereas the normal heart metabolizes a range of substrates, mainly with oxidative metabolism of fatty acids, ischemia drives the myocardium towards relying on anaerobic glycolysis, with reduced BMIPP uptake useful for assessing myocardial viability [89]. Studies have identified an increased incidence of adverse cardiovascular outcomes in patients with HCM and asymptomatic gene mutation carriers who demonstrate reduced BMIPP uptake alongside stress ischemia with 201-thallium SPECT [34]. Furthermore, patients with the obstructive variant of HCM have been noted to have higher degrees of perfusion–metabolism mismatch [30], which may be an important factor to assess post-intervention.

### 4.4. Early Detection of Myocardial Fibrosis

Myocardial fibrosis constitutes a significant irreversible consequence of ischemic and inflammatory processes associated with HCM, correlating with disease severity and an increased risk of adverse outcomes. The development of fibrosis is attributable to underlying chronic ischemia, microvascular dysfunction, or mechanical stress, resulting in collagen deposition and subsequent stiffening of the myocardium [90]. Complications such as arrhythmias, heart failure, and sudden cardiac death have been strongly linked to the presence of underlying fibrosis, which is typically detected in clinical practice through the identification of LGE [91]. However, the fibrosis indicated by LGE represents a late-stage manifestation within the disease continuum.

Advancements in PET radiotracers have facilitated the targeting of proteins that play pivotal roles in the signaling pathways contributing to myocardial fibrosis. Specifically, FAPI serves as a membrane-bound serine protease receptor with minimal basal expression in healthy human tissue, enabling its use in radiotracers employing 18 fluorine or 68 gallium isotopes to localize fibroblast activity accurately [92]. Clinical trials have underscored the pertinence of FAPI-PET imaging, particularly regarding its prognostic implications in HCM. The application of 68-Ga-FAPI PET/MRI has demonstrated a significantly elevated uptake of radiotracer in HCM patients, in contrast with controls, with myocardial segments exhibiting LGE showing the highest levels of 68-Ga FAPI activity [79]. Additionally, heightened FAPI uptake correlates with an increased 5-year risk of SCD [76] and incidence of atrial fibrillation (*p* < 0.05) [79].

Moreover, 18F-FAPI PET/CT has the capability to identify heterogeneous FAPI activity across regions of hypertrophy, sometimes extending beyond these areas. A study conducted by Wang et al. further substantiates the correlation between FAPI uptake and the 5-year risk of SCD (r = 0.32, *p* = 0.03) [76]. An important implication of regions displaying increased 18F-FAPI uptake is the observed reduction in myocardial strain capacity [77]. Given that fibroblast activation precedes the onset of fibrosis, early detection may be achievable through PET imaging of associated signaling processes, thereby enhancing risk assessment strategies for adverse cardiac events.

### 4.5. Evaluation of Ventricular Synchronization

Left ventricular synchrony constitutes a critical factor influencing cardiac efficiency and hemodynamic stability. The presence of dyssynchronous contraction serves as an indicator of deteriorating ventricular function, increased myocardial demand, and heightened risk of arrhythmias [93]. The underlying mechanical mechanisms associated with this abnormal synchronization have been correlated with fibrosis, aberrant conduction pathways, and LVOT obstruction. SPECT has emerged as a non-invasive modality for assessing left ventricular synchrony, potentially in conjunction with simultaneous evaluation of myocardial perfusion and contractility parameters.

The analysis of SPECT facilitates the assessment of phase standard deviation (PSD) and phase histogram bandwidth (PHB), which denote variations in the timing of electrical or mechanical activation during myocardial contraction. Research indicates that PSD and PHB are significantly elevated in patients with HCM who experience major adverse cardiovascular events compared to those without such events (*p* < 0.05) [38]. Consequently, parameters reflecting left ventricular mechanical dyssynchrony hold prognostic value in predicting adverse cardiac outcomes. Furthermore, both SPECT and CMR can be employed in tandem, revealing a strong correlation between PHB, PSD, and the percentage of LGE, thereby highlighting the relationship between dyssynchrony and areas of fibrosis.

Mechanical dyssynchrony of the left ventricle has been associated with parameters such as elevated left ventricular mass and enlarged left atria [36], with a particular association identified with the apical variant of HCM [37]. Other investigation results, such as QRS complex length on ECG and echocardiographic assessment of LV diastolic diameter, have been discussed as predictors of LV dyssynchrony [39]. Studies have also shown greater rates of progression to atrial fibrillation in patients with high degrees of LV dyssynchrony with co-existing left atrial dilatation. Findings derived from studies incorporating SPECT suggest the potential for identifying novel biomarkers indicative of disease progression, which may assist in identifying high-risk HCM patients. Although the role of this has largely been supplanted by echocardiography and CMR, it may retain some utility in evaluating diastolic function in patients with end-stage HCM [94] or predicting complications such as atrial fibrillation [94].

### 4.6. Imaging of Abnormalities in Sympathetic Innervation

Abnormal sympathetic innervation within the myocardium is a significant factor in the pathophysiology of HCM, contributing to an elevated risk of arrhythmias, myocardial dysfunction, and SCD. Iodine-123 metaiodobenzylguanidine (MIBG) has been employed as a radiotracer in SPECT and radionuclide scintigraphy to visualize cardiac sympathetic innervation, particularly in patients experiencing heart failure. In oncology, iodine-123 MIBG has demonstrated high specificity and sensitivity for neuroendocrine tumors [95]. In the context of HCM, myocardial scintigraphy and SPECT have been utilized to evaluate the uptake of I-123 MIBG in regions characterized by ventricular hypertrophy. Indicative markers of abnormal sympathetic innervation include a diminished heart-to-mediastinum (H/M) ratio, an elevated MIBG washout rate [96], and a perfusion–innervation mismatch on SPECT imaging, all of which correlate with an increased risk of arrhythmias and sudden cardiac death.

An early study in 1990 by Nakajima et al. investigated the relationship between regions of augmented perfusion and sympathetic innervation by employing MIBG for sympathetic assessment alongside thallium-201 for blood flow analysis through whole-body scintigraphy and SPECT [97]. The study observed elevated uptake of both tracers in the septa; however, the patterns of I-123 MIBG uptake did not correspond with the distribution of 201-thallium, and the regional MIBG clearance rate in the group with the highest septal thickness (>20 mm) was a significantly higher than the patients with the lowest wall thickness (<16 mm) [97]. This observation underscores the implication of abnormal sympathetic innervation in the myocardium of HCM patients and emphasizes a method for detecting these pathways utilizing nuclear imaging techniques. The use of 123I-MIBG SPECT to assess heart/mediastinal activity has also been carried out, with a higher washout rate observed after delayed imaging in HCM patients. This phenomenon has also been associated with abnormal left ventricular systolic and diastolic parameters, suggesting sympathetic dysfunction as a predictor of mechanical synchrony [20]. High washout rates have also been associated with increased left ventricular mass and maximal wall thickness in a study assessing patients with the specific TPM1-Asp175Asn mutation [21]. Furthermore, the correlation between left ventricular functional reserve and sympathetic nervous activity in patients with non-obstructive HCM can also be explored through scintigraphy both at rest and during dynamic exercise [98]. A linear correlation has been established between the percentage shortening of left ventricular pressure half-time and MIBG clearance, thereby confirming I-123 MIBG as a non-invasive tool for assessing ventricular functional reserve [98].

PET imaging with 18F-fluorodopamine (18F-FDA) and 11C-hydroxyephedrine (11C-HED) radiotracers has also been used to study patterns of abnormal innervation. The uptake of 18F-FDA, when assessed alongside 13N-NH3 PET for myocardial perfusion, showed lower uptake in hypertrophied segments [41]. Similarly, when 11C-HED uptake was observed in conjunction with cardiac MRI and 15-0 labeled water PET, decreased uptake of 18F-FDA and decreased myocardial blood flow was observed in segments displaying late gadolinium enhancement [48]. These studies suggest a mismatch between impaired perfusion and innervation, potentially implicated in the progression of the disease to fibrosis.

### 4.7. Therapeutic and Post-Interventional Imaging

SPECT imaging serves a significant and multifaceted role in post-interventional assessments, particularly in patients following septal ablation. This technique enables the evaluation of myocardial perfusion, functional recovery, and mechanical synchronization parameters. Research conducted by Zhang et al. has shown that in patients undergoing percutaneous transluminal septal ablation, Tc-99m MIBI SPECT imaging reveals a marked decrease in myocardial perfusion within the basal septum during short-term follow-up (*p* < 0.05) [33]. However, evidence suggests a partial recovery in perfusion at mid-term follow-up, thereby underscoring the notion of an adaptive remodeling process occurring post-procedure. Additional findings from the study indicated a significant reduction in LVEF, heterogeneous regional wall motion abnormalities, and a decrease in interventricular septal wall thickness [33]. Beyond perfusion evaluation, phase SPECT analysis offers the capability to assess left ventricular dyssynchrony following alcohol septal ablation. Notably, the analysis displayed a reduction in septal activation delay and an improvement in overall ventricular synchrony among patients identified with baseline dyssynchrony [28]. Furthermore, the measurement of septal–lateral mechanical activation delay has been recognized as a predictor of enhanced left ventricular synchrony post-alcohol septal ablation, demonstrating a sensitivity of 81% and specificity of 88% [28]. This information may aid in selecting appropriate patients and in making prognostic predictions. PET modalities, including 13N-NH3 radiotracer imaging, have also shown utility in measuring improvement in myocardial perfusion post-myectomy. In a 22-patient study conducted by Jorg-Ciopor et al., there was notably improved coronary flow in patients treated surgically compared to medically treated patients [44]. Furthermore, with 15O-water PET, coronary vasodilator reserve has been shown to improve post-septal ablation with a corresponding increase in endocardia/epicardial blood flow ratio [52]. Six months after alcohol septal ablation, the right ventricular oxygen consumption was found to be reduced with 11C-acetate imaging in seven patients [51]. PET modalities may be used to assess the success of procedures in improving perfusion and metabolic efficiency in impaired areas of the myocardium.

### 4.8. Myocardial Energetic Efficiency

11C-acetate PET has been described extensively in the literature for evaluating myocardial energetics in HCM. The radiotracer rapidly enters cardiomyocytes, where it is metabolized, and enters the TCA cycle, leading to oxidative metabolism and production of 11CO2. The clearance rate of 11C-acetate can be used to reflect myocardial oxygen consumption (MVO2). MVO2 can be used to calculate myocardial external efficiency using stroke work (determined through echocardiography or CMR) and heart rate. Studies by Guclu et al. [54] and Witjas-Paalberends et al. [56] investigated patients carrying MYH7 and MYBPC3 mutations who had higher myocardial oxygen consumption but decreased cardiac work compared to controls, suggesting impaired efficiency. This phenomenon has also been observed in patients with the TNNT2 gene mutation [71]. Further studies have revealed greater energetic impairment in patients with obstructive HCM and impaired external efficiency in asymptomatic carriers of gene mutations compared to controls [62]. Recent studies have quantified metabolic efficiency with 13C-acetate PET with the calculation of a work-metabolic index, which was not significantly different in HCM patients and controls at rest, but lower values were identified after acute exercise (*p* = 0.01) [80]. Impaired energetic efficiency may precipitate contractile failure and functional imaging may be used to assess responses to surgical and medical interventions [57].

## 5. Discussion

### 5.1. Summary of Key Findings

Nuclear imaging, particularly PET and SPECT, provides essential insights into the pathophysiological mechanisms associated with HCM, serving as an underappreciated yet significant prognostic indicator of adverse cardiac outcomes. Myocardial perfusion imaging utilizing SPECT facilitates the identification of both fixed and reversible perfusion defects, thereby aiding in the evaluation of ischemic regions within the myocardium. Similarly, PET allows precise quantification of myocardial blood flow, metabolic abnormalities, and LVCD, all of which strongly correlate with diminished myocardial flow reserve and the severity of the disease.

Myocardial fibrosis, which arises from microvascular dysfunction and chronic ischemia, is irreversible and represents the onset of complications such as arrhythmias, heart failure, and SCD. FAPI-PET imaging harnesses the potential to detect pre-fibrotic alterations in the signaling pathways that trigger fibrosis, enabling early detection and timely intervention. Moreover, left ventricular dyssynchrony, a notable characteristic of HCM, can be accurately evaluated through phase analysis of gated SPECT images, offering insights into hemodynamic stability and the risk of systolic and diastolic dysfunction.

**Figure 1:**
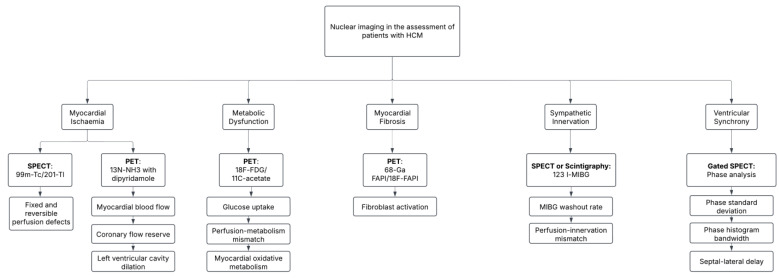
Flowchart outlining the range of potential applications for nuclear imaging in patients with HCM.

Scintigraphy employing radiotracers such as I-123 MIBG provides a distinctive perspective on lesser-known pathological processes, including autonomic dysfunction, which can be instrumental in characterizing the intricate imbalances within the myocardium and examining their effects on overall cardiac functionality. With a wide array of applications and substantial insights into emerging pathological processes, nuclear imaging is crucial in guiding clinical decision-making for high-risk patients. This encompasses assessing the patients’ suitability for interventional procedures, such as alcohol septal ablation (ASA), and monitoring post-procedural outcomes and recovery. These capabilities position nuclear imaging methods as a powerful complementary tool to existing gold-standard modalities such as echocardiography and MRI, offering functional physiological insights that enhance risk stratification and inform management choices. The clinical utility of incorporating nuclear imaging techniques in assessing patients with HCM has been summarized in Figure 1.

### 5.2. Comparison to Conventional Imaging Modalities

The current standard for diagnosing and monitoring patients with HCM predominantly relies on cardiac MRI and echocardiography, as outlined in various clinical guidelines. While both modalities provide precise anatomical detail and essential structural information critical for formulating an HCM diagnosis, nuclear imaging techniques offer the added benefit of delivering real-time insights into the metabolic and functional status of myocardial tissue. Recent literature supports a multimodality imaging approach for patients, as it is essential for comprehensively evaluating their unique disease pathophysiology and can guide the selection of appropriate treatment options [99]. Quantitative imaging of the various discussed pathophysiological processes enables the prediction of disease status and allows for the accurate categorization of a patient’s specific HCM phenotype [100].

#### 5.2.1. Echocardiography vs. Nuclear Imaging

Transthoracic echocardiography continues to be the primary imaging technique for the diagnosis and classification of HCM. This method facilitates real-time evaluation of critical parameters such as wall thickness, outflow tract gradients, and the functionality of the mitral valve. Doppler echocardiography is advantageous in assessing diastolic function, while stress echocardiography aids in provoking obstructive gradients or blunted blood pressure response [101]. However, both modalities are restricted to visualizing the gross anatomical structure of the heart, thereby omitting important details related to microvascular function and subtle ischemic changes. SPECT myocardial perfusion imaging addresses this limitation effectively. The uptake of 99m-Tc or 201-Tl tracers provides insights into the localization of both fixed and reversible perfusion defects, highlighting areas of ischemia that may be susceptible to scarring and fibrosis. Furthermore, nuclear imaging offers a significant distinction regarding the etiology of myocardial malperfusion, particularly in identifying whether obstructive coronary artery disease is the predominant contributing factor. Additionally, the use of 13N-NH3 and rubidium-82 PET serve as valuable complementary methods, allowing for precise quantification of myocardial blood flow with measurable parameters that are more sensitive indicators of microvascular dysfunction than those derived from stress echocardiography.

#### 5.2.2. Cardiac MRI vs. Nuclear Imaging

Cardiac MRI is established in guidelines as the gold-standard method for evaluating cardiac function and myocardial viability (LGE), which is a crucial parameter for the detection of remodeling and arrhythmogenicity. Patients exhibiting > 15% LGE are identified as having a significantly elevated risk of SCD. Consequently, there exists a pressing need to characterize and address these findings early in the disease progression. While LGE serves as a valuable tool for detecting established fibrotic regions, it is concerning that this may represent a late finding associated with a poor prognosis. FAPI-PET imaging mitigates this limitation by identifying fibroblast activation prior to the deposition of extracellular matrix proteins [79]. This capability enables timely recognition and appropriate management of cardiac fibrosis. Notably, FAPI-PET correlates with areas exhibiting LGE on MRI; however, it is also observed in LGE-negative regions where fibrosis may not yet be present, thereby serving as a significant predictor of risk. Furthermore, antecedent processes leading to fibrosis can be elucidated using PET imaging with tracers such as 18-FDG and 11C-acetate, which provide insights into regions exhibiting abnormal ATP metabolism. This offers additional potential biomarkers for evaluating disease severity.

#### 5.2.3. Nuclear Imaging as a Complementary Tool

Echocardiography and cardiac MRI are expected to remain fundamental to the diagnosis of HCM, owing to their widespread accessibility and exceptional ability to provide structural insights necessary for accurate diagnosis. However, nuclear imaging is poised to play a significant role in multi-modal assessment [99]. The increasing adoption of hybrid imaging modalities, such as PET-MRI, exemplifies this trend by integrating functional imaging with precise anatomical details within a single examination.

### 5.3. Role of Nuclear Imaging in Guiding Treatment and Risk Stratification

#### 5.3.1. Risk Stratification Models

The heterogeneous nature of HCM and the severe outcomes associated with the disorder, particularly among the young cohort, necessitates individualized risk assessment and treatment strategies. SCD remains one of the most concerning consequences of HCM, making it imperative to identify and address the risk of SCD in diagnosed HCM patients. Nuclear imaging offers the advantage of providing functional and prognostic markers that may precede structural and symptomatic manifestations. Numerous findings from nuclear imaging scans presented in this paper have demonstrated potential as accurate risk markers in clinical trials, as summarized in Table 3.

#### 5.3.2. Guiding Clinical Decision-Making

Nuclear imaging plays a crucial role in determining appropriate, tailored treatment strategies for patients across various stages of the disease, as well as in monitoring the efficacy of both medical and interventional therapies. As new pharmacological therapies are being investigated for HCM, including myosin inhibitors and metabolic modulators, imaging techniques such as 18F-FDG PET may serve as valuable biomarkers for assessing therapeutic response and myocardial characteristics over time, particularly regarding metabolic dysfunction [51]. Dual imaging with 201-thallium and 123I-BMIPP SPECT may also be utilized to study the effects of metabolic–perfusion matching to assess the efficacy of medical and surgical management [35].

Perfusion imaging with 13N-NH3 PET provides early detection of ischemic changes and enables the identification of high-risk patients before they develop irreversible changes. Whilst earlier studies seem to emphasize the role of SPECT myocardial perfusion imaging in characterizing ischemic patterns and identifying clinical markers of poor prognosis, such as fixed perfusion defects [23], the focus has largely shifted towards the use of stress myocardial blood flow quantification using PET to identify subtle changes such as subendocardial ischemia through transmural perfusion gradients [64]. In clinical practice, biomarkers such as stress-induced left ventricular cavity dilatation show potential as significant indicators of subendocardial ischemia and a greater burden of left ventricular hypertrophy [69]. As the disease process progresses further toward fibrosis, novel markers such as FAPI-PET imaging can identify areas at high risk of fibrosis [76], allowing prognostication and guiding the clinical decision-making process for interventions. Given the correlation between ischemia, fibrosis, and sudden cardiac death across multiple studies, incorporating these findings into current risk models could improve patient outcomes.

For patients with advanced HCM characterized by extensive fibrosis, LVOT obstruction, or ventricular dysfunction, gated SPECT phase analysis can quantify the extent of mechanical dyssynchrony, thereby identifying individuals most likely to benefit from interventions such as alcohol septal ablation [19]. Furthermore, research indicates that patients exhibiting pre-existing left ventricular dyssynchrony can experience significant improvements post-procedure at designated intervals. The identification of septal–lateral mechanical activation delay has emerged as a predictor of successful ablation outcomes [28], thereby underscoring the importance of nuclear imaging in patient selection and outcome prediction. 11C-acetate PET has also been highlighted for its use in evaluating myocardial energetics that could predict contractile function. Myocardial external efficiency levels could be quantified as a potential earlier biomarker of disease progression toward mechanical dysfunction. Multiple studies with PET and SPECT modalities have investigated the burden of specific mutations on structural and functional abnormalities in HCM [49,54,71]. Further exploration of these genotype-phenotype links through functional nuclear imaging and genetic testing could allow the development of comprehensive models of risk stratification and guided management for the range of HCM patients and asymptomatic mutation carriers.

### 5.4. Recent Advancements in Nuclear Imaging Methods

The advent of radiotracers in nuclear medicine has significantly transformed the identification of previously poorly understood pathological processes that materially affect disease outcomes. Emerging methodologies utilizing 82-Rubidium (82-Rb) PET at both rest and stress conditions facilitate the detection of septal ischemia and elucidate the relationship between myocardial perfusion and fibrosis [102]. Furthermore, 11C-KR31173 has been developed for imaging reperfusion post-ischemia, highlighting the regional distribution of myocardial angiotensin II type 1 receptors, which are associated with cardiac remodeling [103]. This approach has predominantly been explored in animal models, along with several human studies, where initial findings indicate an increased distribution of AT1 receptors in the hypertrophied septum of patients with obstructive HCM [65]. Similar observations have also emerged from investigations into non-obstructive HCM [104].

In addition to radiotracers, new biomarkers are being identified to detect irreversible features prior to their onset by targeting precursor molecules. Galectin-3 has been examined as a potential marker for myocardial fibrosis in felines diagnosed with HCM [105]. This particular biomarker is a secretory product of macrophages that promotes pro-fibrotic pathways and stimulates the proliferation of cardiac fibroblasts, and it is linked with aldosterone. Future studies investigating its significance in humans, along with the development of radiotracers targeting this particular molecular pathway, would enhance nuclear imaging techniques aimed at detecting myocardial fibrosis. Furthermore, advancements in nuclear imaging software are progressing. Current technologies may be further refined through the integration of artificial intelligence [106].

### 5.5. Limitations and Future Directions

Despite its numerous advantages, nuclear imaging faces limitations that restrict its widespread application, including high costs, cumbersome protocols, equipment availability, and reader expertise [13]. Importantly, the radiation exposure associated with these techniques is a significant concern, particularly for young adults who may require repeated imaging. Moreover, compared to alternative imaging modalities, such as echocardiography or MRI, nuclear imaging presents challenges in spatial resolution and, thus, commonly results in the underestimation of LVEF.

## 6. Conclusions

HCM is a common genetic disorder accentuated by clinical heterogeneity. Significant adverse complications include heart failure and sudden cardiac death. Although current guidelines primarily utilize echocardiography and CMR for diagnosis and management, substantial gaps persist in comprehending the pathophysiology of the disease. This review highlights the importance of nuclear imaging, which is underutilized in clinical practice and has made notable advancements in recent years, indicating its potential use in the future. PET and SPECT enable the characterization and mapping of critical pathophysiological processes associated with HCM, including microvascular dysfunction, metabolic dysregulation, myocardial fibrosis, and autonomic dysfunction. By complementing traditional imaging techniques, nuclear imaging promotes a more comprehensive approach, offering the development of high-fidelity risk-scoring systems that enhance early recognition, timely management, and risk stratification. As this field continues to evolve, it is essential to encourage further research into the domain of nuclear imaging in HCM through long-term, large-cohort clinical trials with standardized imaging protocols that establish the efficacy of these methods as reliable indicators of disease progression with true clinical utility.

## Figures and Tables

**Table 1:** Key studies involving SPECT in HCM.

Author [Ref]	Year	Outcomes	No. of Patients (Controls)	Nuclear Imaging Type	Results
Keng et al. [19]	2002	Myocardial perfusion post-ethanol septal ablation	30	Thallium 201 SPECT (for patients <200 lb)/Technetium 99 m tetrofosmin (for patients > 200 lb)	SPECT before ablation identified perfusion abnormalities in six patients. Post-septal ablation, SPECT identified the location of infarction with a demonstration of the fixed defects (29/30 patients).
Shimizu et al. [20]	2002	Sympathetic activity	25 (10)	123I-metaiodobenzylguanidine (MIBG) SPECT	There was a notable decrease in heart/mediastinal activity and a higher washout rate of 123I-MIBG 3 h post-uptake in patients with HCM. Uptake patterns showed correlation with left ventricular systolic and diastolic volumes, suggesting sympathetic nervous uptake patterns as a predictor of cardiac dysfunction.
Sipola et al. [21]	2003	Sympathetic activity	21 (9)	(123)I-metaiodobenzylguanidine (MIBG) SPECT	Patients with a TPM1-Asp175Asn mutation had faster 123I-MIBG washout, which was associated with left ventricular mass and maximal wall thickness.
Ward et al. [22]	2003	Apical HCM diagnosis	11 (14)	Dual-isotope rest (thallium 201) and exercise or adenosine stress (technetium 99m tetrofosmin) myocardial perfusion SPECT	A characteristic ‘Solar Polar’ map pattern of intensely bright spots of counts in the apical segment surrounded by a circumferential ring of decreasing counts was identified at rest and on stress SPECT imaging in all apical HCM patients.
Romero-Farina et al. [23]	2004	Myocardial perfusion	101	Myocardial perfusion SPECT	Patients with fixed perfusion defects on SPECT MPI were more likely to suffer from adverse complications of HCM, including death.
Sorajja et al. [24]	2006	Stress MPI	158	Thallium 201 SPECT	Abnormal stress MPI identified in older (>60 years) HCM patients was associated with an increased risk of CV death.
Kawasaki et al. [25]	2007	Stress myocardial perfusion	26	99mTc-tetrofosmin Gated SPECT	Subendocardial ischemia was detected and indexed in all HCM patients with a left ventricular systolic volume cutoff value of 17% identified with good diagnostic value (sensitivity 89%, specificity 82%). Left ventricular cavity dilation was associated with higher index values for subendocardial ischemia.
Cianciulli et al. [26]	2009	Diagnosis of apical HCM	20	Tc-99m sestamibi SPECT with dipyridamole stress	A significant proportion of patients (75%) with apical HCM showed increased tracer uptake and a spade-like deformity of the left ventricle on SPECT.
Baba et al. [27]	2010	Myocardial perfusion	16	Thallium-201 SPECT	Patients with an increased number of myocardial perfusion defects were found to have a higher rate of cardiovascular death, although with no significant difference in the development of associated co-morbidities in a 76-month follow-up period.
Chen et al. [28]	2012	Septal activation and left ventricular dyssynchrony after alcohol septal ablation	32 (28)	Technetium-99m sestamibi SPECT	ASA significantly reduced septal-lateral mechanical activation delay and improved left ventricular synchrony. 12 patients demonstrated significant improvement and 4 with moderate improvement in left ventricular dyssynchrony.
Cocker et al. [29]	2012	Myocardial fibrosis	5	SPECT ^99m^Tc-NC100692	Uptake of ^99m^Tc-NC100692 correlated with late gadolinium enhancement in 3 out of 5 patients.
Utanohara et al. [30]	2012	Myocardial metabolism	146	123I-BMIPP and 201-thallium SPECT	Patients with a metabolism-perfusion mismatch, indicated by decreased BMIPP uptake compared to thallium perfusion, had a greater incidence of adverse cardiac events in a follow-up period of 3.0 ± 1.2 years.
Hashimura et al. [31]	2013	Myocardial fibrosis	20	Technetium-99m MIBI tetrofosmin SPECT, Iodine-123 BMIPP SPECT and Cardiac MRI	Myocardial-to-lumen signal ratios on SPECT were significantly higher in HCM patients (19) with LGE than controls.
Isobe et al. [32]	2014	Mitochondrial dysfunction	20	99mTc-sestamibi SPECT	Patients with an increased washout rate had more disorganized mitochondria with greater ranges of size.
Zhang et al. [33]	2014	Left ventricular myocardial perfusion after alcohol septal ablation	35	Gated Technetium-99m MIBI SPECT	Myocardial septal perfusion and LVEF after ASA were significantly reduced in all patients. Regional wall motion (basal anterior, basal septum, and basal inferior) was significantly decreased following ablation. Thinning of the interventricular septum was apparent after septal ablation.
Takeuchi et al. [34]	2015	Myocardial metabolism and perfusion	35	123I-BMIPP and stress 201-thallium SPECT	In asymptomatic patients with non-obstructive HCM, an increased incidence of adverse cardiovascular events was noted in patients with reduced BMIPP uptake and stress ischemia on thallium SPECT.
Utanohara et al. [35]	2015	Myocardial metabolism and perfusion	125	Dual BMIPP and technetium-99m sestamibi or thallium-201 SPECT	Perfusion-metabolism mismatch was detected in patients with HCM, significantly higher in patients with the obstructive variant. High perfusion-metabolism mismatch scores were correlated with ventricular arrhythmias, potentially advocating for its role as a prognostic marker of SCD.
Ramalho et al. [36]	2016	Left ventricular dyssynchrony	24	Gated MPI SPECT	Patients with a higher left ventricular mass were noted to have more significant mechanical dyssynchrony. Higher degrees of dyssynchrony were correlated with enlarged left atria and atrial fibrillation.
Tsai et al. [37]	2018	Left ventricular dyssynchrony	50	Gated SPECT MPI with thallium-201 and cadmium-zinc-telluride	Patients with HCM were observed to have significant left ventricular dyssynchrony, especially in those with the apical variant. Combining gated SPECT and Doppler echocardiography allowed the detection of fine details of functional changes.
Yuki et al. [38]	2018	Left ventricular dyssynchrony	20	111 MBq 201-Thallium gated myocardial perfusion SPECT	LV dyssynchrony strongly correlated with the extent of LGE, demonstrating the extent of replacement fibrosis.
Ferreira et al. [39]	2019	Left ventricular dyssynchrony	35	Gated-SPECT myocardial perfusion imaging	QRS complex duration on ECG and left ventricular diastolic diameter were identified to be positive predictors of LV dyssynchrony.
Zhou et al. [40]	2019	Diagnosis of apical HCM	22	Gated-SPECT myocardial perfusion imaging	Mean wall thickening and apical hypoperfusion on gated SPECT can be used as a sensitive and accurate tool for diagnosing apical HCM compared to existing cardiac MRI techniques.

**Table 2:** Key studies involving PET in HCM.

Author [Ref]	Year	Outcomes	No. of Patients (Controls)	Nuclear Imaging Type	Results
Li et al. [41]	2000	Myocardial perfusion and sympathetic innervation	8 (15)	13N-ammonia (13NH3) and 6-[18F]-fluorodopamine (18F-FDA) PET	The ratio of 18F-FDA uptake to 13N-ammonia corresponding to perfusion was lower in hypertrophied segments in HCM patients compared to non-hypertrophied areas and controls, suggesting abnormal catecholamine uptake in these areas.
Tadamura et al. [42]	2000	Microvascular dysfunction	12 (6)	13N-NH3 PET with dipyradimole	The patterns of myocardial blood flow and coronary flow reserve observed differed between adults and pediatric patients. Pediatric patients had low flow reserve in hypertrophied segments as well as a marked reduction in septal flow after dipyridamole stress, which was different from the adult patients.
Cecchi et al. [43]	2003	Microvascular dysfunction	51 (12)	13N-NH3 PET with dipyradimole	Patients with reduced myocardial blood flow following dipyridamole infusion were more likely to suffer from adverse consequences such as ventricular arrhythmias. All the patients who died of heart failure and 3/5 who died of sudden death were previously classified to the group with the lowest level of stress MBF.
Jörg-Ciopor et al. [44]	2004	Microvascular dysfunction post-myectomy	22 (15)	13N-NH3 PET with dipyradimole	Observable differences were detected in myocardial blood flow and coronary flow reserve in medically vs. surgically treated patients. Patients who underwent myectomy had impaired coronary flow and reserve compared to controls but higher values compared to medically treated patients.
Knaapen et al. [45]	2006	Microvascular dysfunction	14	Oxygen 15-labeled water PET	Myocardial blood flow at rest was reduced in the ventricular septum compared to the lateral wall. Heterogeneity in delayed contrast enhancement on MRI was associated with impaired perfusion.
Olivotto et al. [46]	2006	Microvascular dysfunction	51 (12)	13N-NH3 PET with dipyradimole	Dipyridamole-induced myocardial blood flow and left ventricular cavity dilatation were identified as predictors of systolic dysfunction (*p* = 0.038 and *p* = 0.031, respectively). In 5 patients who went on to suffer from adverse consequences, including heart failure, dipyridamole-induced myocardial blood flow was particularly low.
Sciagra et al. [47]	2009	Microvascular dysfunction	95	13N-NH3 PET with dipyradimole	HCM patients who had developed atrial fibrillation had a significantly lower stress myocardial blood flow (*p* < 0.0005) and a larger left atrial diameter (*p* < 0.00001).
Gaemperli et al. [48]	2011	Sympathetic activity and microvascular dysfunction	13 (12)	11C-hydroxyephedrine and 15O-labeled water PET	Decreased stress myocardial blood flow and uptake of 11C-hydroxyephedrine were noted in segments displaying late gadolinium enhancement on cardiac MRI.
Olivotto et al. [49]	2011	Microvascular dysfunction	61	13N-NH3 PET with dipyradimole	Patients with mutations in sarcomere myofilament genes had a significant dipyridamole-induced stress myocardial blood flow than patients who were genotype negative.
Timmer et al. [50]	2011	Post-septal ablation myocardial energetics	15	15O-water PET	Coronary vasodilator reserve increased post-septal ablation alongside an increase in endo/epicardial hyperemic myocardial blood flow ratio.
Timmer et al. [51]	2011	Myocardial energetics	21 (11)	11C-acetate PET	Right ventricular oxygen consumption was elevated in 7 HCM patients; 6 months after alcohol septal ablation, the right ventricular oxygen consumption was reduced.
Timmer et al. [52]	2011	Microvascular dysfunction	19 (11)	Oxygen-15 water PET with adenosine stress	Hyperemic myocardial blood flow was decreased in areas of delayed contrast enhancement on cardiac MRI, reflecting severe disease. A correlation was identified between hyperemic myocardial blood flow and contractile function.
Bravo et al. [53]	2012	Microvascular dysfunction and outflow tract obstruction	33	13N-NH3 PET-CT with dipyradimole	No significant correlation was identified between the LVOT gradient and MBF. Maximal wall thickness was identified as the strongest predictor of MBF.
Guclu et al. [54]	2013	Myocardial energetics	23 asymptomatic carriers of HCM genes (14)	11C-acetate PET	Carriers of MYH7 and MYBPC3 mutations had higher myocardial oxygen consumption but lower cardiac work and consequently significant impairment in myocardial external efficiency. Patients with the MYH7 mutation had a steeper reduction in myocardial efficiency compared to carriers of MYBPC3.
Bravo et al. [55]	2013	Microvascular dysfunction	47	13N-NH3 PET-CT with dipyradimole	Delayed enhancement on cardiac MRI is associated with significantly lower stress myocardial blood flow and coronary flow reserve. A subset of patients (*n* = 4) had normal regional perfusion with delayed enhancement on cardiac MRI suggesting other causes other than microvascular dysfunction.
Witjas-Paalberends et al. [56]	2014	Myocardial external efficiency	28 (14)	11C-acetate PET	Myocardial efficiency was lower in patients carrying the MYBPPC3 and MYH7 mutations compared to controls. Specific mutations have consequences for myocardial energetics, which influence the clinical phenotype.
Guclu et al. [57]	2015	Myocardial external efficiency post-myectomy	8	11C-acetate PET	Post-myectomy, there were no identifiable changes in septal or lateral myocardial oxygen intake. Patients showed improvement in regional efficiency of the lateral wall but a decrease in the septum after the procedure.
Bravo et al. [58]	2016	Left ventricular cavity dilatation	61	13N-NH3 PET-CT	Patients with LVCD had greater LV wall thickness but lower stress MBF and CFR. Global peak myocardial blood flow, regional myocardial perfusion abnormalities, and LV thickness were the best predictors of LVCD.
Castagnoli et al. [59]	2016	Microvascular dysfunction	100	13N-NH3 PET-CT with dipyridamole	Patients with lower myocardial blood flow after dipyridamole stress had a sevenfold risk of adverse outcomes including cardiac death. Dipyridamole myocardial blood flow is a significant predictor of outcomes in HCM. Impairment of myocardial blood flow in the lateral ventricular wall reflects non-hypertrophic segment disease and can act as a mortality predictor.
Yalcin et al. [60]	2016	Microvascular dysfunction with dipyridamole stress	104	13N-NH3 PET-CT with dipyridamole	Transmural perfusion gradients were similar at rest between patients with and without LVCD. Myocardial ischemia and diffuse subendocardial hypoperfusion (transmural perfusion gradient) are associated with transient LV cavity dilatation.
Aoyama et al. [61]	2017	Myocardial glucose metabolism	30	18F- FDG PET-CT	18F-FDG uptake was primarily observed in the hypertrophied myocardium in non-obstructive HCM. 18F-FDG uptake was extensive in obstructive HCM, extending beyond the most hypertrophied myocardium. ASA attenuates 18F-FDG uptake (lateral region).
Güçlü et al. [62]	2017	Myocardial efficiency	10 asymptomatic mutation carriers, 10 patients with HOCM (14)	11C-acetate PET-CT	Patients with obstructive HCM had the highest total cardiac oxygen consumption compared to controls and asymptomatic carriers. Asymptomatic carriers had decreased myocardial external efficiency compared to controls. Myocardial external efficiency was not shown to significantly improve in patients with obstructive HCM after surgical myectomy.
Katagiri et al. [63]	2017	Apical HCM diagnosis	34	18F- FDG PET-CT	Spade-shaped FDG uptake in the left ventricular apex may be used to diagnose apical HCM
Sciagra et al. [64]	2017	Microvascular dysfunction with dipyridamole stress	34 (18 preserved LVEF, 16 abnormal LVEF)	13N-NH3 PET with dipyradimole	Subendocardial ischemia, related to TPG, may occur during stress and cause transient LV dysfunction.
Valenta et al. [65]	2017	Myocardial angiotensin II type 1 receptors	4 (4)	11C-KR31173 PET-CT	Myocardial uptake of KR31173 was localized to specific areas of hypertrophy in patients with HCM compared to mild homogenous uptake in controls. There may be increased angiotensin type 1 receptor expression in hypertrophied segments.
Lu et al. [66]	2018	Microvascular dysfunction	133	13N-NH3 PET-CT	Myocardial blood flow heterogeneity index may be increased in patients with HCM who go on to develop ventricular arrhythmias. Elevated stress myocardial blood flow heterogeneity may be used as a biomarker for ventricular arrhythmias and SCD.
Zhao et al. [67]	2018	Microvascular dysfunction	89	13N-NH3 PET	Patients with HCM who had developed pulmonary hypertension had globally reduced stress myocardial blood flow, which was correlated with pulmonary arterial pressures. Myocardial flow reserve was identified as a predictor of pulmonary hypertension in HCM.
Zhao et al. [68]	2019	Microvascular dysfunction	89	13N-NH3 PET-CT	Patients with HCM who had echocardiography identified pulmonary hypertension had significantly reduced stress myocardial blood flow (*p* = 0.024) and myocardial flow reserve (*p* = 0.005).
Lu et al. [69]	2020	Left ventricular cavity dilatation	108	13N-NH3 PET-CT with dipyradimole	PET-LVCD positive group had lower myocardial flow reserve, higher left ventricular mass, and worse GLS at rest/stress, suggesting a greater degree of myopathy and MVD.
Magnusson et al. [70]	2020	Microvascular Dysfunction	25	15O-water, 11C-acetate and 11C-HED PET-CT	The endocardium/epicardium blood flow gradient at stress was lower in patients who suffered non-sustained ventricular tachycardias (NSVT). Mean myocardial oxygen content was elevated in patients with NSVT.
Parbhudayal et al. [71]	2020	Myocardial energetics	14 (14)	11C-acetate PET	Higher MVO_2_ was identified in the septal and lateral walls of the left ventricle in HCM patients compared to controls indicating increased local oxygen consumption. In patients carrying the TNNT2 gene variant, myocardial energetic efficiency was reduced compared to controls.
Calabretta et al. [72]	2022	Microvascular dysfunction	12	13N-NH3 PET-CT	Most patients with apical hypertrophic cardiomyopathy had hypoperfusion identified in the apex of the left ventricle. There were differences identified in myocardial blood flow on stress between the apex.
Wang et al. [73]	2022	Myocardial fibrosis	44	18F-labeled FAPI PET-CT	All HCM patients had FAPI uptake in regions of the left ventricle. Maximal left ventricular thickness, left ventricular outflow tract gradient, and number of hypertrophied segments were all associated with higher FAPI uptake. Patients with an intermediate or high risk of 5-year sudden cardiac death risk scores had higher FAPI uptake (*p* = 0.037). FAPI uptake correlated positively with a 5-year SCD risk score.
Cho et al. [74]	2023	Microvascular dysfunction	50 (20)	C-11 acetate PET	Patients with HCM had lower stress myocardial blood flow and myocardial flow reserve than controls. Myocardial blood flow and mono-exponentially fitted oxidative metabolism positively correlated with myocardial strain on echocardiography.
Svanstroem et al. [75]	2023	Microvascular dysfunction	24	15O-water PET	Sixteen patients had a particular perfusion pattern in the apex or septal wall with increased subendocardial blood flow at rest and reduced myocardial blood flow at rest.
Wang et al. [76]	2023	Myocardial fibrosis	50 (22)	18F-labeled FAPI PET/CT	Increased and intense heterogeneous activity, with FAPI uptake correlating with a 5-year increase in SCD (r = 0.32, *p* = 0.03).
Zhang et al. [77]	2023	Myocardial fibrosis	49	18F–labeled FAPI PET/CT	Intense 18F-FAPI uptake is noted in HCM patients in comparison to controls. 18F-FAPI uptake was associated with strain reduction, and more myocardial regions were detected in comparison to CMR.
Alves et al. [78]	2024	Myocardial glucose metabolism and diastolic dysfunction	30	2-[18F]-FDG PET-CT	A target-to-background ratio of radiotracer uptake >1 was observed in 13.3% of patients. Patients with atrial fibrillation had higher levels of target-to-background ratio of tracer uptake but this was not statistically significant (*p* = 0.07).
Ding et al. [79]	2024	Myocardial fibrosis	20 (11)	[68Ga]Ga-FAPI-04 PET/CMR	[68Ga]Ga-FAPI-04 uptake was significantly higher in HCM patients than controls. FAPI and LGE-positive segments had the greatest myocardial uptake. Higher FAPI uptake was correlated with SCD and AF risk scores.
Wasfy et al. [80]	2024	Myocardial metabolic efficiency	15 (15)	13C-acetate PET	Work metabolic index calculated using indexed stroke volume from dynamic PET images was not significantly different at rest between patients and athlete controls. Results immediately after a 10-minute graded exercise revealed a lower work metabolic index in HCM patients (*p* = 0.01). Acute exercise impaired the myocardial metabolic efficiency in patients with HCM.
Ferreira et al. [81]	2025	Myocardial perfusion and metabolism	30	13N-NH3 and 2-[18F]-FDG PET-CT	Increased 18F-FDG uptake with a target-to-background ratio > 1.1 was detected in 53% of patients. The highest positive correlation with maximal myocardial wall thickness was observed in the anterior wall where there was also a maximum target-to-background ratio of 18F-FDG uptake.

**Abbreviations in Table**: **18F-FDG**—Fluorodeoxyglucose, **18F-FAPI**—Fluorine-18 Fibroblast Activation Protein Inhibitor, **[68Ga]Ga-FAPI-04**—Gallium-68 Fibroblast Activation Protein Inhibitor-04, **13N-NH3**—Nitrogen-13 Ammonia, **AF**—Atrial Fibrillation, **ASA**—Alcohol Septal Ablation, **BMIPP**—Beta-Methyl-P-Iodophenyl-Pentadecanoic Acid, **CMR**—Cardiac Magnetic Resonance, **CFR**—Coronary Flow Reserve, **FAPI**—Fibroblast Activation Protein Inhibitor, **FDG**—Fluorodeoxyglucose, **GLS**—Global Longitudinal Strain, **HCM**—Hypertrophic Cardiomyopathy, **LGE**—Late Gadolinium Enhancement, **LV**—Left Ventricle/Left Ventricular, **LVCD**—Left Ventricular Cavity Dilatation, **LVEF**—Left Ventricular Ejection Fraction, **LVOT**—Left Ventricular Outflow Tract, **MBF**—Myocardial Blood Flow, **MIBI**—Methoxyisobutylisonitrile, **MPI**—Myocardial Perfusion Imaging, **MVD**—Microvascular Dysfunction, **PET**—Positron Emission Tomography, **SCD**—Sudden Cardiac Death, **SPECT**—Single-Photon Emission Computed Tomography, **TPG**—Transmural Perfusion Gradient.

**Table 3:** Potential nuclear imaging biomarkers for SCD risk stratification.

Feature	Potential Risk Markers	Imaging Modality
Myocardial Ischemia	Reduced MBF, impaired CFR, LVCD, subendocardial ischemia	PET (13N-NH3, Rubidium-82), SPECT (99m-Tc)
Metabolic Dysfunction	Increased FDG uptake, reduced acetate clearance, metabolic–perfusion mismatch	PET (18F-FDG, 11C-Acetate)
Myocardial Fibrosis	LGE > 15% LV mass, increased FAPI uptake, early fibroblast activation	PET (68Ga/18F-FAPI), CMR (LGE)
Ventricular Dyssynchrony	Increased PHB, PSD, septal–lateral delay > 100 ms	SPECT (Phase Analysis)
Sympathetic Innervation	Reduced H/M ratio, increased MIBG washout, perfusion–innervation mismatch	SPECT (MIBG Scintigraphy)

## Data Availability

Not applicable.

## Supplementary Materials

The following supporting information can be downloaded at: https://www.mdpi.com/article/10.3390/jcm14072183/s1, Figure S1: PRISMA flow diagram describing the selection of studies for review. Reference [107] is cited in the supplementary materials.

## Abbreviations

The following abbreviations are used in this manuscript:

FDG	Fluorodeoxyglucose
FAPI	Fibroblast Activation Protein Inhibitor
HCM	Hypertrophic Cardiomyopathy
H/M Ratio	Heart-to-Mediastinum Ratio
LVCD	Left Ventricular Cavity Dilation
MBF	Myocardial Blood Flow
MIBG	Metaiodobenzylguanidine
MPI	Myocardial Perfusion Imaging
MRI	Magnetic Resonance Imaging
MYBPC3	Myosin-Binding Protein C
MYH7	Beta-Myosin Heavy Chain
PET	Positron Emission Tomography
PHB	Phase Histogram Bandwidth
PSD	Phase Standard Deviation
SCD	Sudden Cardiac Death
SPECT	Single-Photon Emission Computed Tomography
Tl	Thallium
TNNI3	Troponin I Type 3
TNNT2	Troponin T Type 2
TPM1	Tropomyosin 1
