# Supplementary figures and images for: The Utility of Nuclear Imaging in Hypertrophic Cardiomyopathy: A Narrative Review
Source: J Clin Med. 2025 Mar 22;14(7):2183. doi: 10.3390/jcm14072183 (PMC11989881; doi:10.3390/jcm14072183)

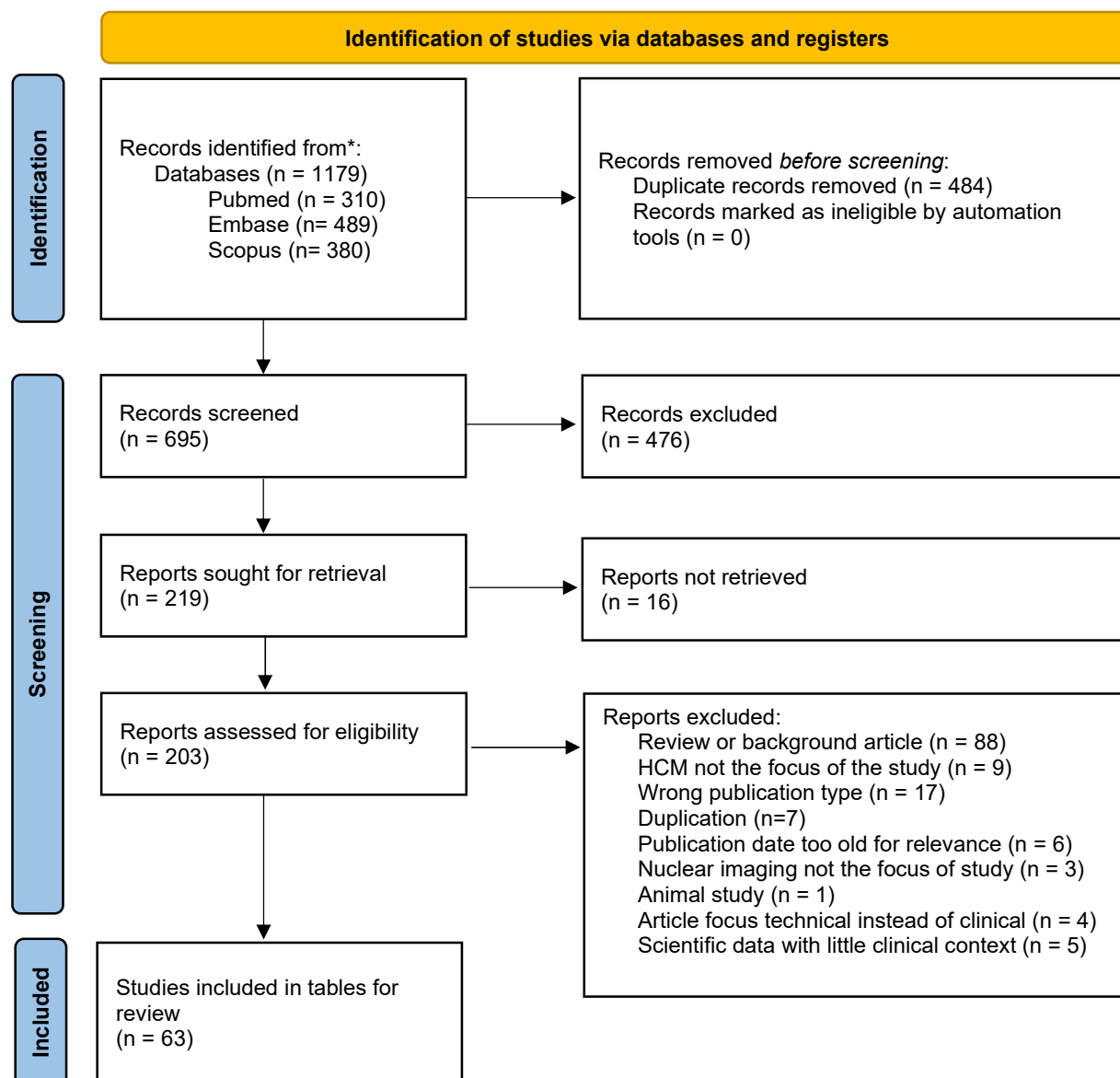

Figure S1: PRISMA flow diagram describing the selection of studies for review.

Supplement: Supplementary file 1 [file jcm-14-02183-s001.zip › jcm-3503276-supplementary.pdf]
